# Longitudinal evaluation of a course to build core competencies in implementation practice

**DOI:** 10.1186/s13012-018-0800-3

**Published:** 2018-08-06

**Authors:** Julia E. Moore, Shusmita Rashid, Jamie S. Park, Sobia Khan, Sharon E. Straus

**Affiliations:** 1 Li Ka Shing Knowledge Institute, St. Michael’s Hospital, Toronto, ON Canada; 2 University of Toronto, Toronto, ON Canada

**Keywords:** Knowledge translation, Implementation, Capacity building, Education, Evaluation, Mixed methods

## Abstract

**Background:**

Few training opportunities are available for implementation practitioners; we designed the Practicing Knowledge Translation (PKT) to address this gap. The goal of PKT is to train practitioners to use evidence and apply implementation science in healthcare settings. The aim of this study was to describe PKT and evaluate participant use of implementation science theories, models, and frameworks (TMFs), knowledge, self-efficacy, and satisfaction and feedback on the course.

**Methods:**

PKT was delivered to implementation practitioners between September 2015 and February 2016 through a 3-day workshop, 11 webinars. We assessed PKT using an uncontrolled before and after study design, using convergent parallel mixed methods. The primary outcome was use of TMFs in implementation projects. Secondary outcomes were knowledge and self-efficacy across six core competencies, factors related to each of the outcomes, and satisfaction with the course. Participants completed online surveys and semi-structured interviews at baseline, 3, 6, and 12 months.

**Results:**

Participants (*n* = 15) reported an increase in their use of implementation TMFs (mean = 2.11; estimate = 2.11; standard error (SE) = 0.4; *p =* 0.03). There was a significant increase in participants’ knowledge of developing an evidence-informed, theory-driven program (ETP) (estimate = 4.10; SE = 0.37; *p =* 0.002); evidence implementation (estimate = 2.68; SE = 0.42; *p <* 0.001); evaluation (estimate = 4.43; SE = 0.36; *p <* 0.001); sustainability, scale, and spread (estimate = 2.55; SE = 0.34; *p <* 0.001); and context assessment (estimate = 3.86; SE = 0.32; *p <* 0.001). There was a significant increase in participants’ self-efficacy in developing an ETP (estimate = 3.81; SE = 0.34; *p <* 0.001); implementation (estimate = 3.01; SE = 0.36; *p <* 0.001); evaluation (estimate = 3.83; SE = 0.39; *p =* 0.002); sustainability, scale, and spread (estimate = 3.06; SE = 0.46; *p =* 0.003); and context assessment (estimate = 4.05; SE = 0.38; *p =* 0.016).

**Conclusion:**

Process and outcome measures collected indicated that PKT participants increased use of, knowledge of, self-efficacy in KT. Our findings highlight the importance of longitudinal evaluations of training initiatives to inform how to build capacity for implementers.

**Electronic supplementary material:**

The online version of this article (10.1186/s13012-018-0800-3) contains supplementary material, which is available to authorized users.

## Background

Implementation science is a rapidly growing field in which significant advances in the methods and effectiveness of implementation interventions have been made [1–3]. To keep abreast with the rapid advances in implementation science, there is a need to train implementers (i.e., those tasked with implementing healthcare evidence) to use the outputs of this science. While capacity building in implementation science is expanding, the targets of these courses are typically researchers and not implementers [4–9]. Courses that target implementers are largely focused on interpreting evidence and facilitating dissemination [10–15]. These courses do not address an expressed need: in one survey of implementers, nearly 80% of respondents wanted to improve knowledge and skills related to implementation [16]. Through surveys, interviews, and an in-person meeting conducted with over 200 Canadian implementers [17], key barriers to applying evidence in practice were lack of organizational capacity (i.e., knowledge and skills) and organizational infrastructure to support implementation. Competency-based training for evidence implementers, which uses consistent standards and objective parameters to assess changes in participants’ knowledge, skills, and outcomes [18, 19], is one approach to meet the capacity demand. When we delivered this course, there were no existing core competencies for the *practice of implementation*. Core competencies for *implementation science* have been identified by various groups, which can serve as the basis for implementation practice core competencies [20–24]. More recently, the National Implementation Research Network developed a working draft of core competencies for an implementation specialist [25].

There is also a gap in the literature describing evaluations of training activities for implementation scientists and practitioners. In response to an editorial in Implementation Science that emphasized the need to rigorously evaluate capacity building initiatives to advance the science and practice of dissemination and implementation [26], several evaluations of training initiatives were published [8, 22, 24, 27]. However, to date, many of these studies evaluated training initiatives that targeted researchers with fewer focused on evidence implementation [28].

Recognizing the need to provide training for implementers and the scarcity of courses, we developed and evaluated the Foundations in Knowledge Translation (KT) Training Initiative [29] and used the findings from its evaluation to inform ongoing KT training initiatives. KT is the term used in Canada to describe dissemination and implementation. We designed the Practicing Knowledge Translation (PKT) course to build on what was learned from the Foundations in KT Training Initiative such as the importance of engaging teams, ensuring opportunities for long-term training and coaching, and tailoring the course to the needs of the team members. Furthermore, for the PKT course, we added modules with more detail on mapping interventions to meet participants’ needs that were identified in the Foundations of KT course. The goal of the PKT course is to provide training in how to use evidence and apply implementation science in healthcare settings. The aim of this study was to describe the PKT course and evaluate participant use of implementation science theories, models, and frameworks (TMFs), knowledge of, self-efficacy in KT, and satisfaction and feedback on the course.

## Methods

### Intervention development

#### Course content

The goal of the course was for participants to increase their use of TMFs, knowledge, skills, and self-efficacy in KT intervention development and implementation. Course content was framed using the knowledge-to-action (KTA) [30] cycle, which guides implementation and incorporates behavior change theories, frameworks, and evaluation models [31]. The KTA cycle was selected because it was developed from a review of more than 30 planned action theories. PKT content was organized into four overarching topics: KT basics, developing an intervention, implementation, and evaluation and sustainability. Since operationalizing the KTA requires adding TMFs, we used Nilsen’s taxonomy as the foundation for understanding and appropriately applying TMFs to each stages of the KTA [31]. When possible, we used TMFs that were developed from reviews of the literature to inform course content. Additional file 1 presents the methods and results to develop core competencies in *implementation practice*, which were used to inform course content.

#### Course structure and delivery

The overall course structure and delivery was informed by behavior change theory (the capability, opportunity, motivation–behavior theory [32]), adult learning theory and practices [33–37], evidence of effectiveness from systematic reviews [38–40], and feedback from previous KT training initiatives [29]. We have provided a summary of course structure and delivery using components of the TIDieR framework [41] (Table 1). More information on the PKT course is publically available online https://knowledgetranslation.net/education-training/pkt/.

**Table 1:** Overview of the PKT course structure and delivery

Intervention component (WHAT)	Mode of delivery (HOW)	Rationale (WHY)
Content presentation	• Delivered over 6 months and included a 3-day in-person workshop and 11 synchronous webinars hosted on WebEx, an online meeting platform. • Instructors used interactive, large-group lectures to present KT theories, models, frameworks, and how to apply these in practice. • Instructors engaged participants in discussion about how the course content related to their own projects.	• Workshop and webinar formats were informed by evidence reviews of continuing education meetings and workshops [39] and internet-based learning in health professions [40]. • Interactive large-group learning components such as enabling participants to add new knowledge to existing knowledge and connect concepts to their own work were informed by transformative adult learning [33] and experiential learning [35] concepts.
Activities	• Individuals were asked to identify their KT learning goals. • Real-time activities during the workshop and webinars were used to encourage individual exploration of KT content. • Some activities were designed as small group work on a common problem and other activities focused on having participants apply content to their own projects. • Self-reflection activities encouraged participants to reflect on progress towards their learning goals.	• Activities provided participants with opportunities to apply and reinforce learning [32]. • Activities were designed based on andragogy principles to enable self-directed learning and increase engagement, understanding, and application of new content [53].
Assignments and feedback	• Participants were asked to complete assignments after each webinar. • Assignments included a knowledge-testing component and an applied component (i.e., participants were asked to apply course content to their projects). • Instructors provided tailored feedback on each assignment to facilitate higher order thinking and application of KT concepts. Assignment assessment and feedback was used to encourage participants to revisit, reflect, and revise their learning.	• Assignments were designed based on concepts of transformative [33] and experiential learning [35]. Feedback was provided based on principles of effective feedback to facilitate adult learning [54].
Implementation facilitator	• Each participant was assigned an implementation facilitator to provide one-on-one support in applying course content to their own work. • The implementation facilitator was available to participants by email and through 3 to 5 h of scheduled one-on-one telephone calls. • The implementation facilitator provided feedback on activities, assignments, and organizational capacity building plans.	• One-on-one learning support was used to facilitate an individualized, supportive learning environment to enhance learner engagement and relevance of course content and increase motivation in applying content [55, 56].
Access to resources	• Participants received a resource package and workbook that included assigned readings, KT resources, and copies of the presented slides. • Canvas [57], an online learning management system, was used to share KT resources, literature, and course content with participants over the 6-month course.	• Easy access to a wide variety of KT resources [40] was used to encourage autonomous and independent learning and continued application of KT content [51, 52].
Social learning opportunities	• In-person and online group discussions facilitated social interaction among participants. • Canvas was used to establish a virtual community to facilitate learner-to-learner and learner-to-instructor engagement and continued communication. • Canvas enabled participants and instructors to post questions, discuss KT topics, and share challenges, successes, and lessons learned.	• Online and in-person discussion and social networking opportunities were informed by social learning theory [58], and best practices in facilitating learner engagement [36, 37].

The course developers and facilitators had a wide range of experience including training in the fields of medicine, public health, adult education; expertise and academic training in conducting quantitative and qualitative research; and practical experience with implementing health and social services with multiple stakeholders in local, national, and international settings (Table 2).

**Table 2:** Overview of the PKT course developers

Course developer	Area of expertise
Dr. Sharon E. Straus	Geriatric medicine, clinical epidemiology, implementation science.
Dr. Julia E. Moore	Implementation science, prevention science, children and youth mental health, psychology.
Sobia Khan	Applied health research, implementation science, public health.

#### Course participants and recruitment

The PKT course was advertised between June and August 2015 using recruitment emails shared with the course developers’ circle of contacts (e.g., implementation researchers, healthcare professionals, project and grant collaborators, participants of previous KT training initiatives). Recruitment ads were posted in online forums and newsletters (e.g., KT Canada Newsletter, Canadian Knowledge Transfer and Exchange Community of Practice, British Columbia Knowledge Translation Community of Practice etc.). Interested participants were invited to complete an application describing their roles, previous experience with implementation, and their interest in participating in PKT. They were also asked to briefly describe project(s) they worked on, their learning goals, and anticipated benefits of participating in PKT. Two course developers reviewed the 19 applications received to assess alignment of the course objectives with applicants’ learning goals and interest in participating, scope and relevance of the identified project(s), and applicants’ position to impact healthcare outcomes. Eligible participants included clinicians, researchers, healthcare managers, and policy makers.

### Evaluation

The PKT course was offered between September 2015 and February 2016 and was assessed using an uncontrolled before and after study design and a process evaluation, using convergent parallel mixed methods [42, 43]. Quantitative and qualitative data were collected concurrently to converge findings and provide a robust analysis.

#### Outcomes

The primary outcome was participants’ use of TMFs in implementation projects. We developed a six-item questionnaire to assess participant use of implementation TMFs (Additional file 2: Part 3—Current KT practice). Each question aligned with one core competency (e.g., use behavior change theory to develop a program). Participants responded to questions using a 5-point scale from “never” to “always.” Secondary outcomes were knowledge and self-efficacy across the six core KT competencies (Additional file 2: Part 1—Knowledge; Part 2—Self-efficacy). These knowledge and self-efficacy questions mapped to the core competencies and indicators (Additional file 1). There were 4 to 11 questions per core competency; mean scores were created for each core competency at each time point. For each knowledge question, participants were asked to rate “I currently have a high level of knowledge in…” for each core competency indicator on a scale from 1 to 7 (strongly disagree to strongly agree). For self-efficacy questions, they were asked to respond to the statement “I am confident in my ability to do the following activities in practice” using a similar Likert scale from 1 to 7. Participants were asked seven questions about course satisfaction (Additional file 2: Part 4—Participant satisfaction).

#### Data collection

Participants completed online surveys on FluidSurveys at baseline, 3, 6, and 12 months after the start of the course to assess use of TMFs, knowledge, self-efficacy, and factors affecting each of these outcomes, using Likert scales from 1 (strongly disagree) to 7 (strongly agree). To maximize response rates, reminders were sent at 1-, 3-, and 7-week intervals after the initial email [44]. Demographic data included the number of years participants had worked in healthcare (Table 3). Participants participated in semi-structured interviews at 3, 6, and 12 months after the start of the course to explore changes in use of TMFs, knowledge, self-efficacy, factors related to use of TMFs, knowledge, and self-efficacy. The semi-structured interview guide was developed to elicit feedback on the participant’s implementation project, use of implementation TMFs, knowledge, self-efficacy, and course satisfaction (see Additional file 3). All interviews were audio recorded and transcribed; transcripts comprised the primary source of data.

**Table 3:** Participant characteristics and demographics

Demographic characteristic (*n* = 15)	*n*	%
Geographic location		
Ontario, Canada	6	40
Alberta, Canada	2	13
British Columbia, Canada	1	7
Ethiopia	2	13
Kosovo	1	7
Saskatchewan, Canada	1	7
Tanzania	2	13
Type of organization		
Healthcare organization	7	47
Government organization	4	27
University	4	27
Years of experience working in healthcare		
More than 10 years	8	53
5–10 years	4	27
1–4 years	1	7
Less than 1 year	2	13

#### Data analysis

Quantitative analyses were conducted using SPSS v 20 (Chicago, IL). To determine if participant outcomes differed over time, multilevel modeling was used with a first-order autoregressive covariance matrix. We used a maximum likelihood estimation model, estimated multiple models to determine the correct error structure using a two-level model with “time” at level 1 and “between-person” variance at level 2; models were chosen based on the Schwarz’s Bayesian criterion. Qualitative analyses were conducted with QSR NVivo 10 using a double-coded, thematic analysis approach [45]. Two independent analysts read four interview transcripts and developed an initial coding tree. Analysts read the four transcripts to familiarize themselves with the data and generated a list of initial codes to apply to the data. This list of codes formed the coding tree, which was then applied to the remaining transcripts, and the coding tree was iteratively refined through discussion. The codebook was systematically applied to the remainder of transcripts, and if any emergent themes appeared in the data, the coding tree was expanded. All data were double coded, and inter-rater reliability between the analysts was calculated using Cohen’s kappa. Any coding discrepancies of between − 1 and 0.6 were discussed and resolved. Once all data were double coded, analysts used charting and visualization tools in NVivo to further explore the data and derive the main themes.

#### Ethics approval

Ethics approval was obtained from the St. Michaels Hospital Research Ethics Board (#15-204). Written consent to participate in the course evaluation was obtained from all participants before course onset. Participation in the course evaluation was voluntary and not connected to course performance; no monetary compensation was awarded.

## Results

### Evaluation

#### Participants

**Fig. 1:**
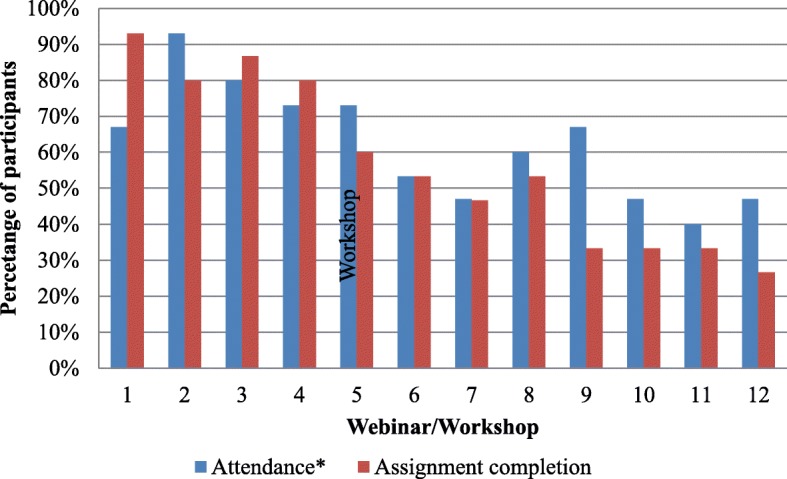
Participants’ attendance and assignment completion over the PKT course duration. *Attendance reflects the number of “live” attendees during a webinar. It is possible that some course participants accessed the webinar recordings post-webinar and viewed them asynchronously; those numbers are not reflected in this figure

Of the 17 participants enrolled in the PKT course, 15 consented to participate in the course evaluation. The majority of evaluation participants had more than 10 years’ experience working in healthcare (53%), were female (73%), and based in Canada (67%) (Table 3). Participants’ attendance in webinars/workshops and assignment completion decreased over the course duration (Fig. 1).

#### Response rates

**Fig. 2:**
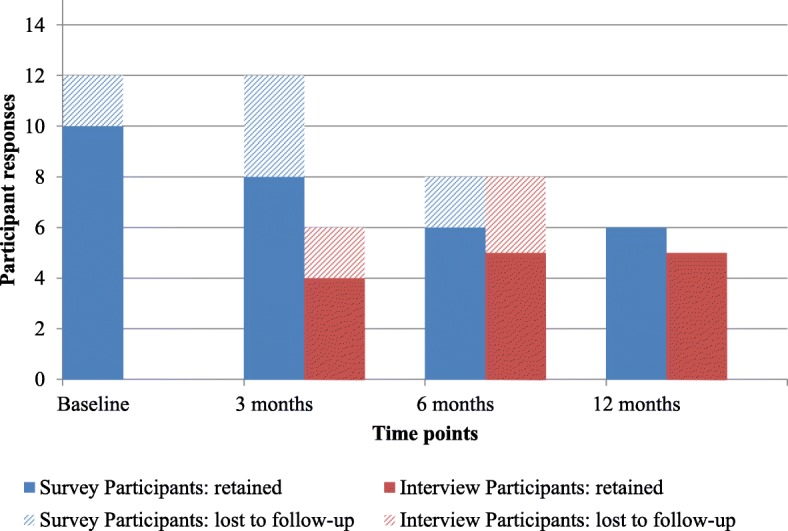
Survey and interview response rates over time

Survey response rates among PKT participants decreased over the four time points; response was 80% at baseline (*n* = 12), 80% at 3 months (*n* = 12), 53% at 6 months (*n* = 8), and 40% at 12 months (*n* = 6). Of the survey participants, 87% completed surveys at two or more time points. Interview participation varied across the three time points: 40% at 3 months (*n* = 6), 53% at 6 months (*n* = 8), and 33% at 12 months (*n* = 5). Of the interview participants, 56% participated in an interview at more than one time point (Fig. 2).

### Change in participants’ use of implementation TMFs

Participants reported an increase in their current use of implementation TMFs (mean = 2.11; estimate = 2.11; standard error (SE) = 0.4; *p =* 0.03). Through the interviews, participants described a change in the way they designed, implemented, and assessed interventions and that they approached these activities in a more rigorous and systematic way since participating in the course (Table 4). At 3 months, some participants described that they sought opportunities to apply KT concepts and use resources provided in the workshop in their work. At 6 months, some participants reported applying TMFs directly in their work. At 12 months, some participants conveyed that they were completing steps of the KTA cycle they had not previously performed (e.g., conducting barrier/facilitator assessments, using frameworks such as the Theoretical Domains Framework and the Consolidated Framework for Implementation Research, developing evidence-informed, theory-driven programs (ETPs) and KT tools etc.). They also described using course resources on a regular basis to inform their work.

**Table 4:** Participants’ change in use of implementation TMFs, knowledge, and self-efficacy over time with examples

Time point	Use of implementation TMFs	Knowledge	Self-efficacy
3 months	“With the course, it’s more about implementation to actually achieve different kinds of results, and so, that’s been very different, and has changed my thinking about how to approach, not only KT, but other… other areas of work.”—P105	“…prior to starting this class, I would not have even known what an ETP [Evidence-informed Theory Driven Program] was. So now not only do I feel like I have a fairly good grasp about what it is, I also am comfortable with certain theories and frameworks related to developing one and I understand the purpose of why we would do that and I also am really interested in learning more about it as well”—P116	“I do not feel confident in that regard, yeah I feel like I need to, one, know more about it, and two, do more like examples or sort of have opportunities for practical application of that”—P116
6 months	“So the PKT course has been beautiful because it enabled that process to move even faster. And I think that’s why it’s one of the most notable things that I got from it, because I can see how it’s changed the way that I approach what I am doing.”—P101	“…the course really helped me step back and say, do not assume we know what the barriers are and start working to overcome them... if we did not do a survey of the people that are supposed to be using this test, we cannot say we truly know why they are or are not. So that was really key learning for me”—P107	“So I guess before I had taken the course I had literally had no confidence in executing anything KT because I basically had no knowledge of it, and so I think I am definitely more confident. I do not think I’d say I would lead …but I feel like I could actively participate on a team” – P117
12 months	“So I have all the course modules printed in binder, all my assignments, it’s all organized, they sit on my desk. I flip through them regularly, I have my favourite chapters that I go to again and again and again and so it’s still fairly fresh.”—P105	“It [KT] just leads to more thought provoking discussions or thoughts at least for me as to what else can I consider.”—P117	“I know what I need to do to have a very effective project. Now my job is to try and find the areas of the organization where it’s possible to actually make it happen.”—P101
Examples of participants’ use of implementation TMFs at an organizational level	▪ Worked on projects where they would offer insights or opinions and try to influence the group to use KT approach. ▪ Developed an overall KT plan for home organization ▪ Compiled and shared KT resources within home organization ▪ Facilitated KT training session with interactive activities for home organization ▪ Established a KT hub or community of practice with researchers, clinicians, and healthcare leaders to share stories, examples, and KT resources ▪ Organized a KT challenge, an opportunity for clinicians and researchers to receive training, facilitation, and funding to evaluate small-scale implementation projects. ▪ Included KT capacity building goals for funding recipients		

Participants described assessing projects underway in their organizations’ to see if projects had been planned and implemented using implementation TMFs. Participants explained that they looked for ways to introduce KT concepts in their organization (e.g., through informal conversations, lunch and learn sessions, workshops). See Table 4 for examples of participants’ use of implementation TMFs at an organizational level.

### Factors influencing participants’ use of implementation TMFs

#### Complexity of KT

Participants said that it was challenging to apply implementation TMFs, since it was a slow, iterative, and complex process. Participants described requiring dedicated time to think through and systematically apply KT content learned through the course. Additionally, they thought their environment was not always conducive to applying KT, as they worked in a healthcare system with constant pressure to deliver quickly, and to make quick decisions and trade-offs to optimize time and resources. In some cases, applying implementation TMFs to projects that were mid-stream required participants to change the original plan (i.e., going backwards before being able to move forward). Participants described KT concepts as being helpful for project planning, but they found practical application to be challenging. Despite these challenges, participants recognized the benefits of applying implementation TMFs and how using KT concepts helped them make progress with their project, which reinforced their intention to use these concepts over time.

#### Organization valuing KT

Using KT concepts within an organization required staff at home organizations, who did not participate in the course, to recognize the value of KT. A few participants mentioned challenges within their organizations, for example, feeling like they were a “lone ranger” as level of receptivity and support for KT varied. Complexity of KT concepts meant it was challenging for participants to find a balance between sharing in-depth information about KT versus providing sufficient information for others to use KT principles. Working with individuals in their organization who were knowledgeable about KT was perceived as being helpful in applying implementation TMFs at an organizational level.

#### Organizational support and resources

Use of implementation TMFs at the organizational level was influenced by the alignment of KT with organizational priorities and interest (e.g., whether the organization prioritized the use of evidence-based KT interventions, placed emphasis on performance measurements and evaluation, had a supportive learning culture emphasizing continued professional development etc.). Some organizations prioritized KT (e.g., were tasked with a provincial agenda to give people KT support as part of their organization’s portfolio). Other participants reported having support from their organization and leadership to use KT approaches, yet did not necessarily feel that there was understanding and support for the time and resources required to build KT capacity and infrastructure to conduct KT activities. Some participants realized that many of their organizations’ projects were focused on and/or were funded for dissemination rather than implementation. Others stated that their organization had competing priorities, such that non-KT projects took precedence.

### Change in knowledge

Across all time points, there was a significant increase in participants’ knowledge in the following core competencies: developing an ETP (estimate = 4.10; SE = 0.37; *p =* 0.002); evidence implementation (estimate = 2.68; SE = 0.42; *p <* 0.001); evaluation (estimate = 4.43; SE = 0.36; *p <* 0.001); sustainability, scale, and spread (estimate = 2.55; SE = 0.34; *p <* 0.001); and context assessment (estimate = 3.86; SE = 0.32; *p <* 0.001; Table 5). Participants did not report an increase in knowledge in the core competency of stakeholder engagement and relationship building. During interviews, participants expressed that the course enabled them to deepen their understanding of KT (Table 4). At 3 months, participants described experiencing a deeper understanding of what KT is and how to approach it, particularly the KT steps. At 6 months, these participants continued to express an increase in knowledge, shifting from a theoretical understanding to a more practical understanding as they were now incorporating KT concepts into their work. They recognized the need to be more systematic in thinking through the complexities and planning for effective implementation. At 12 months, participants emphasized their increased knowledge and described how they better understood the importance of using a theory to underpin activities for behavior change and assessing context, and of developing relationships and engaging end-users when planning for implementation, when compared to course onset.

**Table 5:** Descriptive statistics and fixed effects estimates for knowledge, self-efficacy, KT practice, and satisfaction over time

Outcome	Core competency	Baseline (mean + SE)	3 months (mean + SE)	6 months (mean + SE)	12 months (mean + SE)	*n*	df	*F*	Sig	Estimate	Std error
Use of implementation TMFs		2.11 ± 0.40	2.58 ± 0.40	2.82 ± 0.51	4.38 ± 0.61	14	3 (36.78)	3.39	0.033*	2.11	0.4
Knowledge	Developing an ETP	4.10 ± 0.37	5.20 ± 0.37	5.95 ± 0.42	5.53 ± 0.48	15	3 (23.27)	6.88	0.002*	4.10	0.37
	Implementation	2.68 ± 0.42	4.62 ± 0.42	5.64 ± 0.50	5.41 ± 0.56	14	3 (23.24)	11.54	< 0.001*	2.68	0.42
	Evaluation	4.43 ± 0.36	4.73 ± 0.36	5.98 ± 0.39	6.09 ± 0.43	14	3 (23.36)	10.07	< 0.001*	4.43	0.36
	Sustainability, scale, and spread	2.55 ± 0.34	3.60 ± 0.34	4.73 ± 0.42	5.24 ± 0.47	14	3 (24.50)	9.47	< 0.001*	2.55	0.34
	Context assessment	3.86 ± 0.32	4.91 ± 0.32	5.51 ± 0.36	5.44 ± 0.40	14	3 (23.88)	10.92	< 0.001*	3.86	0.32
	Stakeholder engagement and relationship building	4.77 ± 0.33	5.03 ± 0.33	5.30 ± 0.37	5.36 ± 0.42	14	3 (25.19)	0.86	0.48	4.77	0.33
Self-efficacy	Developing an ETP	3.81 ± 0.34	5.14 ± 0.34	5.41 ± 0.40	5.82 ± 0.45	14	3 (24.68)	9.09	< 0.001*	3.81	0.34
	Implementation	3.01 ± 0.36	4.63 ± 0.36	5.10 ± 0.41	5.42 ± 0.46	14	3 (24.73)	15.5	< 0.001*	3.01	0.36
	Evaluation	3.83 ± 0.39	4.21 ± 0.39	5.33 ± 0.45	5.97 ± 0.50	14	3 (24.68)	6.87	0.002*	3.83	0.39
	Sustainability, scale, and spread	3.06 ± 0.46	3.56 ± 0.46	5.45 ± 0.55	5.30 ± 0.62	14	3 (24.75)	6.24	0.003*	3.06	0.46
	Context assessment	4.05 ± 0.38	4.55 ± 0.38	5.32 ± 0.42	5.42 ± 0.47	14	3 (24.19)	4.22	0.016*	4.05	0.38
	Stakeholder engagement and relationship building	4.49 ± 0.36	4.76 ± 0.36	5.26 ± 0.42	5.47 ± 0.47	14	3 (25.77)	1.34	0.285	4.49	0.36
Satisfaction			6.25 ± 0.20	6.62 ± 0.23	6.63 ± 0.26	12	2 (12.85)	2.08	0.17	6.25	0.2

^*^statistically significant at p < 0.05

### Factors influencing participants’ change in knowledge

During the interviews, a number of participants stated that they had no prior exposure to KT; some said that they had started the course not knowing what KT was, yet knowing that they needed to learn it. Others described having no previous knowledge of KT, yet were able to recognize how some of the concepts overlapped with their previous training and experience in other fields (e.g., continuing professional development, project management, quality improvement, change management, behavior psychology etc.). Most of the participants who were aware of KT mentioned how they were already aware of and/or applying some of these concepts (e.g., using high-quality evidence for program selection, using an evidence-based approach for program implementation, assessing context, and engaging stakeholders etc.) in their work but had not identified these as KT concepts or were more familiar with using other terms to describe these approaches. They were able to link PKT course content to their previous learning, which helped confirm and refine some of the work they were doing. A few participants had background knowledge of KT but described how they thought of KT in a slightly different way at course onset; for example, they had learned about KT from a theoretical perspective and were less familiar with its practical application. Participants who had received previous KT training mentioned that they had not been aware of the distinction between dissemination and implementation, and most of them were more knowledgeable about dissemination than implementation.

### Change in self-efficacy

There was a significant increase in participants’ self-efficacy in developing an ETP (estimate = 3.81; SE = 0.34; *p <* 0.001); implementation (estimate = 3.01; SE = 0.36; *p <* 0.001); evaluation (estimate = 3.83; SE = 0.39; *p =* 0.002); sustainability, scale, and spread (estimate = 3.06; SE = 0.46; *p =* 0.003); and context assessment (estimate = 4.05; SE = 0.38; *p =* 0.016; Table 5). At 3 months, participants indicated that although they were comfortable with course concepts, they required opportunities for application of these concepts to enhance their confidence in applying TMFs. During the 6-month interviews, participants, particularly those who had identified as not having previous KT experience, noted an increase in confidence in their KT work. Some participants expressed that they did not yet feel comfortable leading a KT project on their own but would feel comfortable participating in conversations about KT and contributing ideas in meetings in their home organizations. At 12 months, participants indicated gaining confidence in performing KT activities and feeling like they were prepared to use KT concepts when needed. Participants anticipated gaining experiential self-efficacy through opportunities to apply their course learning to their work (Table 4).

### Factors influencing participants’ change in self-efficacy

Although there was a reported increase in self-efficacy across all core competencies, participants reported needing direction and support from KT experts to reinforce confidence, particularly when building KT capacity in their home organizations. Participants indicated that their confidence in applying implementation TMFs was influenced by their roles (e.g., whether or not they had roles, which involved applying implementation TMFs as part of their daily work), level of experience within their role (e.g., whether they were advanced practitioners or beginners), and scope of practice (e.g., whether they were frontline implementers themselves or responsible for supporting front-line implementers). For example, one participant described how her role was to be a KT resource person for her organization and participate in tasks such as providing feedback on KT sections on grant proposals; she perceived this role helped to reinforce self-efficacy in providing a KT perspective on projects. The opportunity to apply KT concepts to specific implementation projects was similarly perceived to increase participants’ self-efficacy. Participants explained how participating in the course with a specific implementation project was helpful in allowing them to directly apply course concepts. Simple projects with a defined practice change and target audience were perceived to be easier than complex projects that required organizational and system-level change. Since having an active implementation project was recommended but not required for course participants, those participants without implementation projects said it would have been helpful to have an implementation project to make assignments more meaningful. These participants described how using hypothetical examples and hearing about other participants’ projects helped demonstrate how concepts could be applied to the projects they were envisioning. Similarly, exposure to a variety of projects at different stages of implementation, through their own and other participants’ projects, enabled participants to recognize how content could be applied at each stage and across different projects.

### Course satisfaction

Participant satisfaction was high across all time points, means ranged from 6.25 to 6.63 on a 7-point scale. Participants highlighted key strengths of the course including its focus on having a practical lens to implementation, providing access to course facilitators, and the opportunity to network and learn from others. Participants expressed that they were highly satisfied with both the content and the format of the course, as the course allowed them to learn and feel prepared and confident to practice KT. They described that a key highlight of the course was the facilitators’ encouragement throughout the course and the use of examples and analogies to explain complex concepts so that they were understandable and applicable.

Participants reported that the webinars and bi-weekly assignments helped them to stay abreast with course content, although this was reported by those who continued to complete webinars and assignments. The online learning component was perceived to provide easy access to course materials. The course materials were described as being very helpful and a good balance between in-depth theory and practical examples. Access to an implementation facilitator was perceived to have been a critical support for directly applying implementation TMFs to participants’ own work. Participants appreciated feedback on assignments, particularly input on their ETP development and KT plans. Qualitative data on participant satisfaction is presented in Additional file 4.

Participants shared suggestions for improving the course (Table 6). Since delivering and evaluating this iteration of the PKT course, we delivered the course 7 times with an effort to address these improvement suggestions. Changes included an increased focus on engaging stakeholders, building relationships and gaining implementation buy-in. We encouraged applicants from the same organizations to participate together to optimize impact within the organization. Within the larger workshops, we organized participants at similar levels and roles (e.g., policy makers vs. researchers vs. health care managers etc.) for small group activities to foster discussions based on collective experiences. We continue to identify ways to increase the affordability and sustainability of this course, such as providing online courses.

**Table 6:** Examples of suggestions for improvement for PKT course content and components

Components	Suggestions
Content	• Compare KT concepts and terminology with terminology from other fields (e.g., change management, quality improvement, project management) to highlight where the concepts relate/differ. • Course places more emphasis on targeting individual change than change at an organization or system level. • Reinforce some concepts and provide additional opportunities to practice and receive feedback on certain concepts (e.g., mapping, developing logic models, and motivational interviewing). • Provide more training on developing skills and best practices related to facilitation others in the use of implementation TMFs. • Balance level of detail consistently across different topics areas (e.g., course spent more time on implementation but less time on dissemination, evaluation, and sustainability). • Consider introducing evaluation and sustainability earlier to start thinking about how to plan for these in the beginning. • Use one example throughout to help consolidate understanding; also have diversity of examples so there is a balance between clinical, public health, and policy level examples
Webinars, activities and assignments	• Increase engagement of participants during webinars by having each participant take turns in sharing an overview or examples from their own projects, present a recap of a topic area, share thoughts on assigned readings, or share rationale behind answers during webinar activities • Increase opportunities to interact with other participants via online learning platform, Canvas, by including group assignments where participants have the option of pairing up or working in small groups on some assignments, increase facilitated discussion posts where participants are asked to reflect on a specific topic or reading • Share reminders/prompts about webinar and assignment schedule including specific dates during each webinar and on Canvas. • Establish and maintain community of practice among course participants to share resources, support, project success stories, and lessons learned beyond the end of the course.
Facilitation	• Have more structured implementation facilitation and systematic way of supporting learners by identifying a facilitator at the beginning of the course and clarify facilitator’s role to ensure regular/periodic facilitation support throughout the duration of the course, having designated time to meet with facilitator during in-person workshop to describe project and gain specific guidance on applying content, shifting office hours to scheduled facilitation sessions to increase participants’ likelihood of accessing facilitation support. • Have follow-up check-in support 1 year post-course.

## Discussion

Six core competencies were used to develop, deliver, and evaluate the PKT course (see Additional file 1). Participation in the PKT course was associated with increased use of implementation TMFs, knowledge, and self-efficacy over the duration of the course, and these changes were sustained 12 months after course onset. After the course was completed, we observed statistically significant differences in all measured core competencies except stakeholder engagement and relationship building. We likely did not see a change in stakeholder engagement and relationship building because this was the least covered topic in the course with the least amount of concrete approaches that result in positive outcomes. Our observed outcomes were influenced by factors such as participant’s role in their organization, stage and scope of their projects, and level of organizational and leadership support for using KT.

Findings from this evaluation are comparable with other studies evaluating dissemination and implementation training initiatives and identified a number of factors that can be leveraged to enhance future initiatives for implementers [22, 24, 27]. Course facilitators can consider participants’ previous experience with KT (e.g., beginner, intermediate, advanced) [46] and fit with participants’ previous learning and experience from other fields (e.g., terminology and concepts from physician education, project management, quality improvement, change management, behavior psychology etc.). Building upon previous knowledge can help scaffold and reinforce learning [34, 47]. To help increase self-efficacy in KT, training initiatives can link to participants’ scope of practice (e.g., whether they are intervention developers, frontline implementers, or responsible for supporting front-line implementers) and opportunities to apply KT concepts. This is consistent with findings from other evaluations of dissemination and implementation research training programs [2]. In addition, experiential learning techniques such as problem-based learning can be leveraged to maximize skill transfer [48, 49], and implementation researchers and practitioners appreciate an emphasis on the application of theories through problem-based scenarios [27]. This research is reflected in PKT course participants’ reports that it was helpful to have their own project to work on throughout the course.

Consistent with other studies evaluating capacity building programs [6, 24, 27], PKT participants valued informal opportunities to network with other implementers and navigate through challenges associated with using TMFs (e.g., how to identify and select appropriate TMFs, when and how to use them etc.) with others in the course. Several participants continued to connect and meet, and there was a course participant who switched organizations to work with another course participant. These networking opportunities can be used to leverage the knowledge and skills acquired through the course. Similar to other studies that have assessed the use of mentorship approaches in implementation science capacity building initiatives [6, 46, 50], PKT participants valued having access to an implementation facilitator to provide support in applying course content to their work through regular check-ins and feedback.

This evaluation was unique in that we assessed reported use of TMFs at the organizational level, going beyond individual measures of knowledge and self-efficacy. Although all participants had some level of organizational support (i.e., the organization paid for them to attend), there was variability in organizational learning and absorptive capacity [51, 52]. The absorptive capacity of the organizations (including organizational priorities, organizational readiness, and buy-in) in combination with the participants’ organization roles (e.g., being responsible for KT, having the influence or capacity to transform learning and processes embedded within the organization) affected participants’ experiences in championing KT within the organization. These findings have implications for which participants/organizations should be accepted and for organizations in how they support participants after PKT.

Some limitations should be noted. First, we had a small sample size; the number of course participants was intentionally small to allow for individualized learning, support, and coaching. Second, survey and interview respondents’ response rates decreased over time. Not all participants completed the surveys or interviews. It should be noted that the response rate was highest at the 3-month time point, suggesting there was greater engagement immediately after the workshop. It is possible that participants who were less engaged in the course did not participate in the assignments, surveys, and interviews; as a result, their perspectives and outcomes were not captured. There was also attrition in survey and interview participation over time due to changes in participant’s role in their organization or changes in their employment status. Third, we used a quasi-experimental research design without the use of a control group. For this reason, causal inferences regarding predictors and outcomes cannot be made. Fourth, our data were collected using self-report measures, some of which had not been assessed for validity, specifically those measuring knowledge, self-efficacy, and use of TMFs. However, the survey results were consistent with the interview results. Fifth, we assessed outcomes at the level of individual course participants; outcomes at an organizational level warrant further exploration.

As healthcare systems seek new approaches to increase capacity in effective evidence-based implementation, the PKT course can be used as a model to inform how to develop, deliver, and tailor continuing professional development initiatives to help build capacity at individual and organizational levels. The core competencies identified for the practice of implementation can help organizations and decision makers assess and monitor competencies of practitioners so they can identify and address capacity gaps and needs to enhance their implementation efforts. Next steps for future research include developing a valid and reliable measure of practitioners’ knowledge and self-efficacy and adapting valid, reliable tools to measure change in behavior, intention to use, and actual use of implementation science.

## Conclusion

Process and outcome measures collected over 12 months indicated that participation in the PKT course increased participants’ knowledge, self-efficacy, and use of implementation TMFs and the factors that moderated these outcomes such as participation in a network of learners. Our findings highlight the importance of conducting longitudinal evaluations of training initiatives to help inform how to build capacity for implementers.

## Additional files

Additional file 1: Implementation practice core competencies. (DOCX 22 kb)

Additional file 2: PKT evaluation survey. (DOCX 550 kb)

Additional file 3: PKT evaluation interview guide. (DOCX 29 kb)

Additional file 4: Course satisfaction—qualitative data. (DOCX 14 kb)

## Abbreviations

ETP: Evidence-informed, theory-driven program
KT: Knowledge translation
KTA: Knowledge-to-action
PKT: Practicing knowledge translation
SE: Standard error
TMFs: Theories, models, and frameworks
