# Supplementary material for: Longitudinal evaluation of a course to build core competencies in implementation practice
Source: Implement Sci. 2018 Aug 6;13:106. doi: 10.1186/s13012-018-0800-3 (PMC6080520; doi:10.1186/s13012-018-0800-3)
Supplement: Supplementary file 1 — Implementation practice core competencies. (DOCX 22 kb) [file 13012_2018_800_MOESM1_ESM.docx]

**Additional file 1. Implementation practice core competencies**

*Methods*

To identify implementation practice core competences, we reviewed existing competencies and elicited feedback from experts. Building on the work by Padek and colleagues, we identified four manuscripts that described competencies for dissemination and implementation research [20, 26-28]. In collaboration, two authors (SK, JEM) reviewed all competency domains and indicators identified across the four studies (e.g., “theory and approaches”; “design and analysis”), removed those relevant only for researchers (e.g., “design and analysis”) and combined similar competencies across sources. Competency domains were the higher level constructs describing thematic areas; indicators described knowledge, skills and behaviours related to each of these domains. Through this process, we identified a preliminary list of six core competencies (designing programs; implementation; evaluation; sustainability, spread, and scale up; diagnosis; stakeholder engagement and relationship-building) for implementation practice. Drawing from the indicators described in the four manuscripts, we matched relevant indicators to the six core competencies and revised the language to reflect the target audience for the training, namely, implementation practitioners. We identified 26 indicators (e.g., use an implementation framework to plan for implementation) for achieving these competencies (Table 1)*.*

We invited experts in the science and practice of implementation to provide feedback on the competencies and indicators in a modified Delphi with an online survey created using FluidSurveys. Experts were identified through our circles of contact; specifically, Canadian and US researchers/implementers who had received peer-reviewed funding to conduct implementation research or had experience in evidence implementation in health care. Using a 7-point Likert scale, experts indicated their level of agreement on whether each competency: was fundamental to/benefited implementation practice and enabled implementers to meet their goals; and should be included, discarded, or restructured. They then rated the importance of each of the indicators (7-point Likert scale: 1= not at all important and 7= very important) and provided open-text comments on the competencies and indicators. Qualitative feedback was consolidated and when two experts made comments on the same competency or indicator, we reviewed all responses and revised the item to align with the responses.

*Results*

Eleven experts responded to the survey (four Canadians; seven Americans) to provide feedback on implementation practice core competencies and indicators. Respondents recommended restructuring one competency (designing programs), renaming one competency (diagnosis to context assessment), and adding one competency (dissemination). Median ratings for all indicators were ≥6. Most recommendations related to language and the inclusion of 5 additional indicators (e.g., planning for dissemination by selecting appropriate messages and dissemination strategies). We revised the list of core competencies and indicators based on this feedback, and the final list included seven core competencies (i.e., designing programs; implementation; evaluation; sustainability, spread, and scale up; context assessment; stakeholder engagement and relationships-building; and dissemination) and 29 indicators (Table 1). As the course focused on implementation, we excluded “dissemination” and the associated 3 indicators and addressed 6 of the 7 competencies and 26 of the 29 indicators.

**Table A1.** Core competencies and indicators for implementation practice

| Core Competency | Indicators |
| --- | --- |
| 1.0 Designing Evidence-based, Theory-driven Programs (ETPs): Competency in this category includes the knowledge and skills required to design an ETP by assessing needs, using behaviour change constructs and theory, appraising evidence, mapping appropriate strategies to both theory and evidence, and understanding implementation, evaluation, sustainability and stakeholder engagement principles. | - Understand levels of evidence and how to appraise the strength and quality of evidence - Conduct needs assessment and prioritize gaps - Assess barriers and facilitators to change - Link barriers and facilitators to behaviour change theory - Select implementation strategies that target barriers and facilitators to change using behaviour change theory - Develop a logic model for the ETP |
| 2.0 Implementation: Competency in this category includes the knowledge and skills to plan and implement an ETP at the individual, organizational, community and systems levels. This includes processes and contexts of implementation, and logistical considerations for ETP rollout. | - Use an implementation framework to plan for implementation - Assess readiness for change in implementation settings - Assess the implementation context - Tailor implementation strategies to the implementation context - Evaluate adaptations and their potential impact on outcomes - Develop an implementation team |
| 3.0 Evaluation: Competency in this category involves the knowledge and skills to plan and interpret evaluation mechanisms to monitor implementation and assess outcomes. | - Evaluate implementation quality - Use an evaluation framework to guide evaluation - Understand the aims of process, outcomes and impact evaluations and the application of these evaluation types in implementation - Select appropriate evaluation indicators. |
| 4.0 Sustainability, Scale-up and Spread: Competency in this category involves the knowledge and skills to plan for and assess the sustainability of ETPs, and for scaling up and spreading ETPs. | - Understand factors related to sustainability, spread and scale-up of ETPs outlined in various theories and frameworks - Develop a sustainability plan - Create implementation supports for spread and scale-up |
| 5.0 Context assessment: Competency in this category includes the ability to assess situations and contexts that may affect implementation before, during and after ETP rollout. This includes skills that enable the practitioner to identify root causes of ambivalence/resistance to change and the impact of internal and external implementation contexts on implementation. It also includes the skills to be able to facilitate resolution of identified issues. | - Identify and explore adaptive challenges to implementation - Understand the values, loyalties, losses, and benefits of different approaches to a variety of adaptive challenges |
| 6.0 Stakeholder Engagement: Competency in this category includes the knowledge and skills necessary to actively engage key stakeholders throughout the implementation process, and to form and sustain positive and productive collaborations. | - Identify all key stakeholders, define the nature of their stake in the change and determine their level of buy-in for the change - Know methods of engaging and involving stakeholders at all key points of ETP implementation (planning, implementation, evaluation, sustainability), to incorporate stakeholder interests in the process - Mediate between different interests of stakeholders using skills such as team building, negotiation, conflict management and group facilitation to build partnerships in pursuit of a common goal - Engage (or encourage others to engage) in action planning to resolve anticipated and unanticipated implementation issues - Develop strategies for overcoming stakeholder ambivalence or resistance to change |
| 7.0 Dissemination: Competency in this category includes the skills required to translate and re-format evidence to enhance communication about research findings and/or the ETP to the end-user. | - Communicate complex information to various end-user groups in appropriate formats, considering suitability and readability of content. - Plan for and carry out dissemination initiatives by selecting key messages and appropriate dissemination strategies. - Develop knowledge tools for various end-user groups. |
