# Supplementary material for: Longitudinal evaluation of a course to build core competencies in implementation practice
Source: Implement Sci. 2018 Aug 6;13:106. doi: 10.1186/s13012-018-0800-3 (PMC6080520; doi:10.1186/s13012-018-0800-3)
Supplement: Supplementary file 2 — PKT evaluation survey. (DOCX 550 kb) [file 13012_2018_800_MOESM2_ESM.docx]

**Additional file 2: PKT Evaluation Survey**

Practicing Knowledge Translation:  End of Course Evaluation Survey

# Thank you for agreeing to respond to the Practicing Knowledge Translation (PKT) course evaluation survey.

We are soliciting feedback from PKT course participants at four (4) different points in time throughout the duration of the course to:

1. Evaluate course outcomes

- Whether the PKT course changes knowledge of KT practice
- Whether the PKT course changes confidence in participants’ abilities to practice KT (self-efficacy)
- Behaviours related to KT practice (current behaviours, intentions to change behaviour and KT practice behaviours performed since the end of the course).

1. Evaluate the process of the course

- Whether the PKT course was delivered with quality
- Whether participants were satisfied by the PKT course offerings
- General feedback on strengths and areas of improvement.

**This is the last of four surveys. This survey is meant to collect data on knowledge, self-efficacy and behaviours related to KT practice. We are also interested in evaluating the process of the course**. The survey should take no more than 30 minutes to complete.

If you have any questions or technical difficulties, please direct inquiries to Shusmita Rashid (rashids@smh.ca)To start the survey, click on the “next” button, below.

*Note that surveys cannot be saved. Please arrange to respond to and submit the survey in one 30-minute time period*.

Before you start, we would like to know a bit more about you.

# Please enter your participant I.D. in the box below.

# Please indicate the number of years you have been working in healthcare.

|  | Less than 1 year |
| --- | --- |
|  | 1- 4 years |
|  | 5-10 years |
|  | More than 10 years |

# Part 1: Knowledge

In this section, you are presented with a series of statements related to your **current knowledge** on aspects of KT practice. The statements are divided into 6 parts based on the KT core competencies. Developing an evidence-informed, theory-driven program (ETP) Implementation Evaluation Sustainability, scale and spread Diagnosis Stakeholder engagement and relationship building Note that in Part 2 we will ask you about your confidence in your ability to do these activities in practice.Please rate your level of agreement with each statement on a scale from 1 (strongly disagree) to 7 (strongly agree). If this knowledge is not applicable to your work please select ‘N/A’.

# Developing an evidence-informed, theory-driven program (ETP)

I currently have a high level of knowledge in:

|  | 1 Strongly disagree | 2 | 3 | 4Neither agree or disagree | 5 | 6 | 7 Strongly agree | N/A |
| --- | --- | --- | --- | --- | --- | --- | --- | --- |
| Understanding levels of evidence to support decision making |  |  |  |  |  |  |  |  |
| Appraising the quality of evidence |  |  |  |  |  |  |  |  |
| Identifying gaps in practice and behaviour |  |  |  |  |  |  |  |  |
| Key behaviour change theories |  |  |  |  |  |  |  |  |
| Key frameworks for behaviour change |  |  |  |  |  |  |  |  |
| Conducting a barriers and facilitators assessment |  |  |  |  |  |  |  |  |
| Interpreting a barriers and facilitators assessment |  |  |  |  |  |  |  |  |
| Mapping barriers to a selected theory and strategies to establish program activities that comprise the ETP |  |  |  |  |  |  |  |  |

# Part 1: Knowledge (continued)

Please rate your level of agreement with each statement on a scale from 1 (strongly disagree) to 7 (strongly agree). If this knowledge is not applicable to your work please select ‘N/A’.

# Implementation

I currently have a high level of knowledge in:

|  | 1 Strongly disagree | 2 | 3 | 4Neither agree or disagree | 5 | 6 | 7 Strongly agree | N/A |
| --- | --- | --- | --- | --- | --- | --- | --- | --- |
| Implementation models and process frameworks |  |  |  |  |  |  |  |  |
| Assessing readiness for change in implementation settings |  |  |  |  |  |  |  |  |
| Interpreting readiness for change assessment findings to tailor ETPs to diverse contexts. |  |  |  |  |  |  |  |  |
| Roles of implementers and recipients of implementation strategies at all levels of implementation. |  |  |  |  |  |  |  |  |

# Part 1: Knowledge (continued)

Please rate your level of agreement with each statement on a scale from 1 (strongly disagree) to 7 (strongly agree). If this knowledge is not applicable to your work please select ‘N/A’.

# Evaluation

I currently have a high level of knowledge in:

|  | 1 Strongly disagree | 2 | 3 | 4Neither agree or disagree | 5 | 6 | 7 Strongly agree | N/A |
| --- | --- | --- | --- | --- | --- | --- | --- | --- |
| Types of implementation quality (e.g., dose, fidelity, participant responsiveness) |  |  |  |  |  |  |  |  |
| Evaluation of implementation strategies |  |  |  |  |  |  |  |  |
| Aims of process evaluations |  |  |  |  |  |  |  |  |
| Aims of outcomes evaluations |  |  |  |  |  |  |  |  |
| Aims of impact evaluations |  |  |  |  |  |  |  |  |
| Selecting appropriate evaluation indicators. |  |  |  |  |  |  |  |  |
| Qualitative methods, and the appropriateness of using these methods to assess evaluation indicators. |  |  |  |  |  |  |  |  |
| Quantitative methods, and the appropriateness of using these methods to assess evaluation indicators. |  |  |  |  |  |  |  |  |
| Strengths and limitations of evaluation designs. |  |  |  |  |  |  |  |  |

# Part 1: Knowledge (continued)

Please rate your level of agreement with each statement on a scale from 1 (strongly disagree) to 7 (strongly agree). If this knowledge is not applicable to your work please select ‘N/A’.

# Sustainability, scale and spread

I currently have a high level of knowledge in:

|  | 1 Strongly disagree | 2 | 3 | 4Neither agree or disagree | 5 | 6 | 7 Strongly agree | N/A |
| --- | --- | --- | --- | --- | --- | --- | --- | --- |
| Factors related to sustainability of ETPs outlined in various theories and frameworks. |  |  |  |  |  |  |  |  |
| Factors related to spread of ETPs outlined in various theories and frameworks. |  |  |  |  |  |  |  |  |
| Factors related to scale up of ETPs outlined in various theories and frameworks. |  |  |  |  |  |  |  |  |
| Strategies to sustain implementation |  |  |  |  |  |  |  |  |

# Part 1: Knowledge (continued)

Please rate your level of agreement with each statement on a scale from 1 (strongly disagree) to 7 (strongly agree). If this knowledge is not applicable to your work please select ‘N/A’.

# Diagnosis

I currently have a high level of knowledge in:

|  | 1 Strongly disagree | 2 | 3 | 4Neither agree or disagree | 5 | 6 | 7 Strongly agree | N/A |
| --- | --- | --- | --- | --- | --- | --- | --- | --- |
| Factors that can create individual and organizational ambivalence or resistance to change. |  |  |  |  |  |  |  |  |
| Factors that may act as opportunities or facilitators for change. |  |  |  |  |  |  |  |  |
| Social networks as they relate to impacting change in an implementation setting |  |  |  |  |  |  |  |  |
| Professional dynamics as they relate to impacting change in an implementation setting |  |  |  |  |  |  |  |  |
| Needs and responsibilities of different stakeholder groups. |  |  |  |  |  |  |  |  |
| External implementation contexts as challenges or catalysts for change. |  |  |  |  |  |  |  |  |
| Strategies that attempt to mitigate or resolve issues that are internal or external to the implementation setting. |  |  |  |  |  |  |  |  |

# Part 1: Knowledge (continued)

In this section, you are presented with a series of statements related to your **current knowledge** on aspects of KT practice.Please rate your level of agreement with each statement on a scale from 1 (strongly disagree) to 7 (strongly agree). If this knowledge is not applicable to your work please select ‘N/A’.

# Stakeholder engagement and relationship building

I currently have a high level of knowledge in:

|  | 1 Strongly disagree | 2 | 3 | 4Neither agree or disagree | 5 | 6 | 7 Strongly agree | N/A |
| --- | --- | --- | --- | --- | --- | --- | --- | --- |
| Different stakeholder types. |  |  |  |  |  |  |  |  |
| Foundations of team building, negotiation, conflict management and group facilitation to build partnerships and strong relationships. |  |  |  |  |  |  |  |  |
| Foundations of team building to build partnerships and strong relationships. |  |  |  |  |  |  |  |  |
| Foundations of negotiation to build partnerships and strong relationships. |  |  |  |  |  |  |  |  |
| Foundations of conflict management to build partnerships and strong relationships. |  |  |  |  |  |  |  |  |
| Foundations of group facilitation to build partnerships and strong relationships. |  |  |  |  |  |  |  |  |
| Communicating information to various end-user groups |  |  |  |  |  |  |  |  |
| Interpreting information for various end-user groups. |  |  |  |  |  |  |  |  |
| Selecting appropriate formats for communication, considering suitability and readability of content. |  |  |  |  |  |  |  |  |
| Planning for dissemination of key messages. |  |  |  |  |  |  |  |  |
| Selecting appropriate dissemination and communication strategies. |  |  |  |  |  |  |  |  |

# Part 2: Self-Efficacy

In this section, you are presented with a series of statements on your **self-efficacy** related to aspects of KT practice. The statements are divided into 6 parts based on the KT core competencies. Developing an evidence-informed, theory-driven program (ETP) Implementation Evaluation Sustainability, scale and spread Diagnosis Stakeholder engagement and relationship buildingPlease rate your level of agreement with each statement on a scale from 1 (strongly disagree) to 7 (strongly agree). If this statement is not applicable to your work please select ‘N/A’.

# Developing an evidence-informed, theory-driven program (ETP)

I am confident in my ability to do the following activities in practice:

|  | 1Strongly disagree | 2 | 3 | 4Neither agree or disagree | 5 | 6 | 7 Strongly agree | N/A |
| --- | --- | --- | --- | --- | --- | --- | --- | --- |
| Understand levels of evidence used to support decision making |  |  |  |  |  |  |  |  |
| Appraise the quality of evidence |  |  |  |  |  |  |  |  |
| Identify gaps in practice and behaviour |  |  |  |  |  |  |  |  |
| Apply behaviour change theories to ETP planning |  |  |  |  |  |  |  |  |
| Use behaviour change frameworks in ETP planning |  |  |  |  |  |  |  |  |
| Conduct a barriers and facilitators assessment |  |  |  |  |  |  |  |  |
| Interpret a barriers and facilitators assessment |  |  |  |  |  |  |  |  |
| Map barriers to a selected theory and strategies to establish program activities that comprise the ETP |  |  |  |  |  |  |  |  |

# Part 2: Self-Efficacy (continued)

Please rate your level of agreement with each statement on a scale from 1 (strongly disagree) to 7 (strongly agree). If this statement is not applicable to your work please select ‘N/A’.

# Implementation

I am confident in my ability to do the following activities in practice:

|  | 1Strongly disagree | 2 | 3 | 4Neither agree or disagree | 5 | 6 | 7 Strongly agree | N/A |
| --- | --- | --- | --- | --- | --- | --- | --- | --- |
| Use implementation models and frameworks to guide implementation planning |  |  |  |  |  |  |  |  |
| Assess readiness for change in implementation settings |  |  |  |  |  |  |  |  |
| Interpret readiness for change assessment findings to tailor ETPs to diverse contexts |  |  |  |  |  |  |  |  |
| Identify the roles of implementers and recipients of implementation strategies at all levels of implementation |  |  |  |  |  |  |  |  |

# Part 2: Self-Efficacy (continued)

Please rate your level of agreement with each statement on a scale from 1 (strongly disagree) to 7 (strongly agree). If this statement is not applicable to your work please select ‘N/A’.

# Evaluation

I am confident in my ability to do the following activities in practice:

|  | 1Strongly disagree | 2 | 3 | 4Neither agree or disagree | 5 | 6 | 7 Strongly agree | N/A |
| --- | --- | --- | --- | --- | --- | --- | --- | --- |
| Apply the types of implementation quality (e.g., dose, fidelity, participant responsiveness) to full-scale implementation. |  |  |  |  |  |  |  |  |
| Incorporate/plan process evaluation for implementation. |  |  |  |  |  |  |  |  |
| Incorporate/plan outcomes evaluation for implementation. |  |  |  |  |  |  |  |  |
| Incorporate/plan impact evaluation for implementation. |  |  |  |  |  |  |  |  |
| Select appropriate evaluation indicators. |  |  |  |  |  |  |  |  |
| Design evaluations using qualitative data collection and analysis methods. |  |  |  |  |  |  |  |  |
| Design evaluations using quantitative data collection and analysis methods. |  |  |  |  |  |  |  |  |
| Interpret evaluation findings by considering the strengths and limitations of evaluation designs. |  |  |  |  |  |  |  |  |

# Sustainability, scale and spread

I am confident in my ability to do the following activities in practice:

|  | 1Strongly disagree | 2 | 3 | 4Neither agree or disagree | 5 | 6 | 7 Strongly agree | N/A |
| --- | --- | --- | --- | --- | --- | --- | --- | --- |
| Apply sustainability principles to implementation planning. |  |  |  |  |  |  |  |  |

# Part 2: Self-Efficacy (continued)

Please rate your level of agreement with each statement on a scale from 1 (strongly disagree) to 7 (strongly agree). If this statement is not applicable to your work please select ‘N/A’.

# Diagnosis

I am confident in my ability to do the following activities in practice:

|  | 1Strongly disagree | 2 | 3 | 4Neither agree or disagree | 5 | 6 | 7 Strongly agree | N/A |
| --- | --- | --- | --- | --- | --- | --- | --- | --- |
| Identify and probe for individual and organizational ambivalence or resistance to change. |  |  |  |  |  |  |  |  |
| Identify and probe for opportunities or facilitators for change. |  |  |  |  |  |  |  |  |
| Identify social networks as they relate to impacting change in an implementation setting. |  |  |  |  |  |  |  |  |
| Identify professional dynamics as they relate to impacting change in an implementation setting. |  |  |  |  |  |  |  |  |
| Identify needs and responsibilities of different stakeholder groups. |  |  |  |  |  |  |  |  |
| Identify external implementation contexts as challenges or catalysts for change. |  |  |  |  |  |  |  |  |
| Select and directly use, or encourage others to use, strategies that attempt to mitigate or resolve issues that are internal or external to the implementation setting. |  |  |  |  |  |  |  |  |

# Part 2: Self-Efficacy (continued)

Please rate your level of agreement with each statement on a scale from 1 (strongly disagree) to 7 (strongly agree). If this statement is not applicable to your work please select ‘N/A’.

# Stakeholder engagement and relationship building

I am confident in my ability to do the following activities in practice

|  | 1Strongly disagree | 2 | 3 | 4Neither agree or disagree | 5 | 6 | 7 Strongly agree | N/A |
| --- | --- | --- | --- | --- | --- | --- | --- | --- |
| Identify all key stakeholders, define the nature of their stake in the change and determine their level of buy-in for the change. |  |  |  |  |  |  |  |  |
| Use team building skills to build partnerships and strong relationships. |  |  |  |  |  |  |  |  |
| Use negotiation skills to build partnerships and strong relationships. |  |  |  |  |  |  |  |  |
| Use conflict management skills to build partnerships and strong relationships. |  |  |  |  |  |  |  |  |
| Use group facilitation skills to build partnerships and strong relationships. |  |  |  |  |  |  |  |  |
| Mediate between different interests of stakeholders in pursuit of a common goal. |  |  |  |  |  |  |  |  |
| Communicate with and interpret information for various end-user groups. |  |  |  |  |  |  |  |  |
| Select appropriate formats for communication, considering suitability and readability of content. |  |  |  |  |  |  |  |  |
| Plan for dissemination of key messages. |  |  |  |  |  |  |  |  |
| Select appropriate dissemination and communication strategies. |  |  |  |  |  |  |  |  |

# Part 3: Current KT Practice

In this section, you are presented with a series of statements related to your **current** KT practice.Each statement taps into a key practice that should be performed when developing and implementing an evidence-informed, theory-driven program. Rate how frequently you have performed these behaviours to date, on a scale from 1(never) to 5 (always). Select N/A if you have never had the opportunity to engage in the listed practice.

# When an evidence-based program does not exist for a behaviour/practice change I am targeting in my KT project, I:

|  | 1Never | 2Rarely | 3Sometimes | 4Very Often | 5Always | N/A |
| --- | --- | --- | --- | --- | --- | --- |
| Use behaviour change theory to develop a program. |  |  |  |  |  |  |
| Develop a program that incorporates both clinical/policy/population evidence that supports a practice and that supports the implementation strategy for that practice. |  |  |  |  |  |  |

# When my KT project involves implementing an ETP, I:

|  | 1Never | 2Rarely | 3Sometimes | 4Very Often | 5Always | N/A |
| --- | --- | --- | --- | --- | --- | --- |
| Tailor, or help others to tailor, a program to the local context, while considering core components. |  |  |  |  |  |  |
| Use an implementation process/ framework to develop, or help others to develop, an implementation plan. |  |  |  |  |  |  |
| Include, or help others to include, implementation quality measures in program evaluation. |  |  |  |  |  |  |
| Develop, or help other to develop, a sustainability plan. |  |  |  |  |  |  |

Since taking the PKT course, please identify which of these activities you have engaged in:

[Applying KT questions]

Using KT theories, models and frameworks in a new project

Using KT theories, models and frameworks in an existing project

Using PKT materials (e.g., slides, activity sheets, resources) in your implementation project

[Training others in KT questions]

Sharing PKT course materials (e.g., slides, activity sheets, resources) with members within your organization.

Sharing PKT course materials (e.g., slides, activity sheets, resources) with members from external organizations.

Training people in your organizations on how to apply KT theories, models and frameworks

# Part 4: Implementation Quality and Participant Satisfaction

In this section, you are presented with a series of statements related to the implementation quality of the course as well as your satisfaction with the course components. Rate your level of agreement with the statements below from 1 (Strongly Disagree) to 7 (Strongly Agree).

# Implementation Quality and Participant Satisfaction

|  | 1Strongly disagree | 2 | 3 | 4Neither agree or disagree | 5 | 6 | 7 Strongly agree | N/A |
| --- | --- | --- | --- | --- | --- | --- | --- | --- |
| I was extremely satisfied with the session readings and resources |  |  |  |  |  |  |  |  |
| Overall, I was satisfied with the presentations |  |  |  |  |  |  |  |  |
| Overall, I was satisfied with how the content applies to my work |  |  |  |  |  |  |  |  |
| I was satisfied with the session activities |  |  |  |  |  |  |  |  |
| I was satisfied with the format of the session (presentation, group activity etc.) |  |  |  |  |  |  |  |  |
| I was satisfied with the content of the sessions |  |  |  |  |  |  |  |  |
| Overall, I thought the course was implemented with high quality |  |  |  |  |  |  |  |  |

# 1. Please list the three things you liked best about the course:

# 2. Please list the three ways in which the course could be improved:

# 3. Please provide any other comments:
