# Supplementary material for: Longitudinal evaluation of a course to build core competencies in implementation practice
Source: Implement Sci. 2018 Aug 6;13:106. doi: 10.1186/s13012-018-0800-3 (PMC6080520; doi:10.1186/s13012-018-0800-3)
Supplement: Supplementary file 3 — PKT evaluation interview guide. (DOCX 29 kb) [file 13012_2018_800_MOESM3_ESM.docx]

**Additional file 3: PKT Evaluation Interview Guide**

**End of Course Interview Guide for PKT Course Participants (~ 45- 60 minutes)**

1. Introduction:

Hello [*insert name of course participant*], my name is XXX and I am a research assistant with the Knowledge Translation Program at St. Michael’s Hospital. Thank you very much for agreeing to participate in this interview and taking the time to speak with me.

2. Purpose of study and interview:

As you may recall, the Practicing Knowledge Translation (PKT) course is an intensive training program in Knowledge Translation (KT) offered by the KT Program at St. Michael’s Hospital to individuals who are looking to build capacity in, and a culture of, KT in their home organizations. The purpose of this study is to evaluate the *outcomes* of the PKT course by assessing the extent to which it enhances participants’ knowledge of KT theory and practice, self-efficacy in implementing KT, and behavior. We will also evaluate the *process* of the PKT course by assessing implementation quality of the course and participants’ satisfaction.

We wanted to take this opportunity to speak with you as one of the participants of the course to learn about your experience with the course. The information we discuss today will be considered for ongoing improvements in the course design.

3. Structure of the Interview Process:

We will start off by briefly going over the terms of consent, after which I will ask for your verbal consent to participate in today’s interview. The terms of consent outlined today were also included in the hard copy of the consent form that you completed at the start of the course.

During the interview I will be asking you questions about your knowledge of KT theory and practice, self-efficacy in implementing KT, and behavior related to KT practice. I will also ask you questions about your experience with the course (e.g., things you liked versus things that could be improved). The interview will take approximately 45 minutes.

The results of today’s interview will help us better understand your experience evaluate *outcomes* and *process* of the PKT course. The results of today’s interview will also help us to evaluate the course components and make improvements in the course design. Do you have any questions at this point? [*Address any concerns; if none, continue*].

4. Consent

*Outline the terms of verbal consent.*

I will now go over the terms of consent:

- Your participation in this study is voluntary.
- You can choose to not participate or you may withdraw at any time, even after the interview has started.
- This interview is confidential
- The interview will be recorded.
- The audiotape will be transcribed and names will be removed as it is being transcribed.
- Once the transcribed information has been assessed for accuracy by the study coordinator, the audiotape will be erased.
- The interview will be analyzed by an independent analyst.
- The results will be aggregated and reported anonymously. The results may be used in presentations and publications.

At this point, do you have any questions?

I will now get started by turning on the recorder and asking you, in turn, to state your verbal consent to participate in today’s interview. [TURN ON AUDIO RECORDER]

Today is [*insert date*] and I am interviewing PKT Course Participant [insert participant ID]; Do you consent to being interviewed and recorded today?

5. Start the interview

|  | **Interview Question**  So, this interview will be very similar to the last one (the mid-course interview), so I’m going to ask you all of the questions, but if you already expressed everything you wanted to last time, that’s fine, or you can feel free to re-iterate. First I would like to ask you about your knowledge and self-efficacy related to the specific KT competencies covered in the course. Then later, I will ask you about your thoughts on the content and format of the course. |
| --- | --- |
|  | ***Knowledge and Self-Efficacy*** |
| 1. | Do you think the PKT course has changed your **knowledge** about KT?  (*If yes*) Can you provide me some examples of how your knowledge has changed?   - (*If they struggle to remember*) Let me refresh your memory. The course covered the following core competencies for the practice of KT:   - Developing an evidence-informed, theory-driven program (ETP) *(how to develop an ETP),*   - Implementation *(how to plan and implement an ETP at the individual, organizational, community and systems levels).*   - Context Assessment *(how to identify factors in the internal and external implementation contexts which may affect implementation)*   - Stakeholder engagement *(how to actively engage key stakeholders throughout the implementation process)*   - Dissemination *(how to translate and re-format evidence to enhance communication about research findings and/or the ETP to the end-user)*   - Evaluation *(how to monitor implementation and assess outcomes)*   - Sustainability, scale and spread *(how to plan for and assess the sustainability of ETPs, and for scaling up and spreading ETPs).*   Do you think you have more or less knowledge about any of these 4 key topics?  (*If yes*) Can you provide me some examples of how your knowledge has changed? |
| 2. | Do you think the PKT course impacted your **self-confidence** with practicing KT?  (*If yes*) Can you provide me some examples of how your self-confidence has changed?  *(if they struggle to understand the question)* Do you feel you have an increased or decreased ability to practice KT since taking the PKT course?  *(still struggling)* Do you think you could develop an ETP after taking the course?  (*If yes*) Can you provide me some examples of how you feel you could practice KT?  (*If no*) Can you explain why you feel that way?   - In relation to 4 KT core competencies:   - Developing an evidence-informed, theory-driven program (ETP),   - Implementation,   - Context Assessment, and   - Stakeholder engagement   Please provide examples. |
|  | ***KT Practice Behaviour*** |
|  | The following questions relate to how participating in the course may have influenced your thinking and behavior (or planned behavior), in relation to KT practice. |
| 3. | Since participating in the PKT course, has your **behaviour** changed in terms of KT practice?  (*If yes*) Can you provide me some examples of how your behavior has changed?  (*If they struggle to remember*) Let me refresh your memory. The course covered the following areas:  Developing an Evidence-informed, theory-driven program (ETP):  conducting needs assessments  assessing & mapping barriers and facilitators to theoretical frameworks  selecting and defining implementation strategies  developing logic models  Implementation planning:  considering the implementation context  stakeholder engagement  developing and working with implementation teams  tailoring ETPs  Evaluation, sustainability and dissemination:  implementation quality  monitoring implementation,  evaluating ETPs  sustaining ETPs  dissemination  Have you had an opportunity to apply the concepts learned through the course to your work? If so, please provide examples.  If your behaviour in relation to any of these components remain unchanged, do you have plans to change them in the future (i.e., in your next project)? Why or Why not? |
|  | ***PKT Course Feedback*** |
| 9. | This question was asked during the last interview too, but we wanted to give you a chance to either reiterate or let us know if your thoughts have changed, and to give feedback on the last few lectures. If you have no additional things to tell us about any of these topics, that’s okay.  What are your thoughts on the format of the PKT course?   - What are your thoughts about the online learning experience? - What are your thoughts about the 3-day workshop experience? - What are your thoughts regarding using Canvas as the online learning platform? - Probes:   Can you think of anything that would have made your learning experience more enjoyable? |
| 10. | What are your thoughts on the content of the course?   - Were there other topics you would like to cover in the course? - Have the assignments facilitated your learning of KT theories and practices? Why or why not? - Probes: Do you have additional feedback on the final assignment you completed recently? - Do you feel that the online activities were useful in terms of building your understanding about KT? Why or why not? - Probes: Are there specific lectures, activities, assignments, or handouts/resources that stick out to you as particularly useful? - Probes: Are there specific lectures, activities, assignments, or handouts/resources that stick out to you as particularly not useful? |
| 11. | Do you feel that the course has enabled you to meet your KT learning goals?  Please provide some examples.   - Probes: - Do you feel that your KT learning goals have changed as a result of taking this course? - Are there other ways in which the PKT course could help address your ongoing KT learning goals? |
| 12. | In general, what are some things you liked about the course?   - Please provide examples.   What are some things that could be improved upon?   - Please provide examples.   Do you have any ideas of ways that you could be supported after the course ends?  Probes: email updates, access to other participants, if materials remain accessible |
| *Do you have any additional comments that you would like to add?* | |

Wrap Up

Thank you for your time today.
