# Supplementary material for: Longitudinal evaluation of a course to build core competencies in implementation practice
Source: Implement Sci. 2018 Aug 6;13:106. doi: 10.1186/s13012-018-0800-3 (PMC6080520; doi:10.1186/s13012-018-0800-3)
Supplement: Supplementary file 4 — Course satisfaction—qualitative data. (DOCX 14 kb) [file 13012_2018_800_MOESM4_ESM.docx]

**Additional file 4: Course Satisfaction – Qualitative Data**

| Qualitative data |
| --- |
| “*I think this course gives you the facility to take the knowledge and actually apply it in a systematic way to assist people in not just creating implementation plans, but trouble shooting, diagnosing... it doesn’t matter where you hit the implementation process. I can hit it mid-stream, end-stream, sustainability stream... I can hit it wherever it is on the map and I can still facilitate and help and I think that’s a huge advantage that I got from the course that I didn’t have before*.” – P101 |
| “*I think it [the course] makes it really easy to interact and have dialogue and rather than it be just sort of a lecture, it’s more like a learning experience, so that was really helpful, it makes it really easy to learn… this facilitated participant enthusiasm … which I found to be very contagious, the more participants started integrating [implementation] frameworks and theories into their projects and then seeing results from that was really inspiring and really sort of helped solidify stuff for me*.” – P116 |
| “*Every single webinar had us doing interactive things, and if it hadn’t been for that, it’s much too easy to get distracted when you’re sitting in a conference room on a webinar… So, not only … did it help me learn by actually doing an exercise on the webinar, it definitely helped me stay engaged*.” – P107 |
| “*So having a mentor meant that out of that whole sea of faces there was one person I could call or email with any question... then I got specific feedback about my project which is very helpful* – P107 |
